# Characteristics, management, and outcomes of patients with VIPoma–A retrospective analysis of the ENETS database

**DOI:** 10.1111/jne.70219

**Published:** 2026-06-23

**Authors:** Detlef K. Bartsch, L. Mais, L. de Mestier, E. Baudin, G. Kaltsas, J. Hofland, R. Garcia‐Carbonero, E. Tiensuu Janson, M. G. McNamara, M. Andreassen, S. Grozinsky‐Glasberg, F. Spada, H. Leupe, F. Panzuto, M. Fleschen, H. Klinkhammer

**Affiliations:** ^1^ Department of Visceral‐, Thoracic‐ and Vascular Surgery Philipps University and UKGM GmbH Marburg Germany; ^2^ Department of Medical Oncology Hôpital Edouard Herriot Lyon France; ^3^ Service de Pancreato‐Gastroenenterologie Beaujon Hospital, Université Paris‐Cité Clichy France; ^4^ Département d'Imagerie, Cancérologie Endocrinienne Gustave Roussy Villejuif France; ^5^ 1st Department of Internal and Propaedeutic Medicine, EKPA LAIKO ENETS and EURACAN 4 Center of Excellence National and Kapodistrian University of Athens Athens Greece; ^6^ Department of Internal Medicine, Section of Endocrinology ENETS Centers of Excellence Erasmus MC Cancer Institute Rotterdam Netherlands; ^7^ Oncology Department Hospital Universitario 12 de Octubre, IIS Imas12, Facultad de Medicina, Universidad Complutense de Madrid (UCM) Madrid Spain; ^8^ Department of Medical Sciences, Endocrine Oncology, and Science for Life Laboratory Uppsala University Uppsala Sweden; ^9^ Department of Oncology University of Manchester/The Christie NHS Foundation Trust Manchester UK; ^10^ Department of Nephrology and Endocrinology Rigshospitalet, and ENETS Center of Excellence Rigshospitalet University Kopenhagen Copenhagen Denmark; ^11^ Neuroendocrine Tumor Unit, Division of Medicine and Faculty of Medicine the Hebrew University, Hadassah Medical Center Jerusalem Israel; ^12^ Division of Gastrointestinal Medical Oncology and Neuroendocrine Tumors European Institute of Oncology (IEO), IRCCS Milan Italy; ^13^ Department of Nuclear Medicine University Hospitals Leuven Leuven Belgium; ^14^ Department of Imaging and Pathology University of Leuven Leuven Belgium; ^15^ Department of Medical‐Surgical Sciences and Translational Medicine, Sapienza University of Rome, Digestive Disease Unit Sant'Andrea University Hospital Rome Italy; ^16^ ENETS Office Eurropean Neuroendocrine Tumor Society e.V. Berlin Germany; ^17^ Institute for Medical Biometry and Statistics University of Marburg Marburg Germany

**Keywords:** ENETS Center of Excellence, ENETS database, functional pancreatic neuroendocrine tumor, VIPoma

## Abstract

Introduction: VIPoma is an extremely rare functioning pancreatic neuroendocrine tumor. Therefore, data regarding treatment and outcome are very limited. Aim (s): This multicenter study aimed to analyze clinical characteristics, real‐world management, and outcomes of patients with VIPoma. Methods: Patients with VIPoma treated in a 20‐year period at 14 referral centers for neuroendocrine tumors (NET) were collected in the ENETS Database. Clinical characteristics, therapeutic interventions, and outcomes were analyzed retrospectively. Disease‐free survival (DFS) and overall survival (OS) were estimated using the Kaplan–Meier method. Results: Of 70 patients, 59 (54% male) with a median age of 55 years were included. Forty‐six (78%) patients were diagnosed due to the classical watery diarrhea–hypokalemia–achlorhydria (WDHA) syndrome, and 40 (68%) presented with distant metastases at diagnosis. Fifty‐two (88%) had serum VIP levels >2 times the upper normal limit and the median Ki‐67 index was 5% (range 1%–40%). Surgery of the primary VIPoma was performed in 32 (54%), with curative intent in 22 (37%). In patients with stage I–III disease (*n* = 19), long‐term cure was achieved in only 26% (5/19), with a median DFS of 81 (95%‐CI: 1–215) months and 10‐year OS of 78% (95%‐CI: 61%–100%). In stage IV patients (*n* = 53, 40 at diagnosis, 13 disease progression), the sequence and type of 354 treatment lines varied, while SSA (*n* = 77, 22%), loco‐regional liver‐directed therapy (*n* = 72, 20%), and chemotherapy (*n* = 69, 19%) were most often applied. Median OS for patients with stage IV at diagnosis was 142 (95%‐CI: 87–not available (NA)) months with 10‐year survival of 50% (95%‐CI: 35%–73%). Conclusion: Patients with VIPoma treated at NET centers have relatively favorable survival, even with distant metastases at diagnosis, although long‐term cure rates remain low.

## 1 INTRODUCTION

Vasoactive intestinal peptide–secreting tumors (VIPomas) are exceptionally rare functioning pancreatic neuroendocrine tumors (PanNET), first described by Verner and Morrison in 1958.^1^ The annual incidence is estimated to be approximately 1 in 10 million people, accounting for about 1%–3% of all PanNET.^2^, ^3^, ^4^ VIPomas are characterized by excessive secretion of vasoactive intestinal peptide (VIP), resulting in the classical watery diarrhea–hypokalemia–achlorhydria (WDHA) syndrome.^1^ The associated fluid and electrolyte disturbances may be life‐threatening and often necessitate urgent medical care. VIPomas occur sporadically in about 95% and in the context of multiple endocrine neoplasia type 1 (MEN1) syndrome in 5%–14%.^4^, ^5^

More than 95% of VIPomas arise in the pancreas, although they have historically also been reported in the colon, lung, esophagus, jejunum, and liver.^2^, ^6^ At diagnosis, at least 50% of patients have primary tumors >5 cm in size, which either cause the WDHA syndrome or local symptoms or even both, with a prevalence (>50%) of distant metastases at diagnosis, particularly to the liver.^2^, ^3^, ^4^, ^5^ Elevated plasma VIP levels are highly suggestive for VIPoma, but can very rarely also be encountered in neurogenic tumors including ganglioneuroblastomas, ganglioneuromas, and pheochromocytomas.^6^, ^7^

Surgical resection remains the only potentially curative treatment for VIPoma.^2^, ^8^, ^9^ However, due to an advanced stage at diagnosis, curative surgery is feasible in only a minority of patients. In metastatic disease, treatment aims to control both tumor progression and hormone‐mediated symptoms. Somatostatin analogues (SSA) have long stood as the cornerstone of antisecretory therapy, while systemic chemotherapy, molecular targeted therapies, loco‐regional liver‐directed treatments, peptide receptor radionuclide therapy (PRRT), as well as debulking surgery are used to control the VIPoma and its metastases growth as well as the WDHA syndrome.^2^, ^8^, ^9^

Because of the extreme rarity of VIPoma, evidence guiding specific treatment decisions is very limited to small retrospective series and case reports.^3^, ^4^, ^10^, ^11^, ^12^, ^13^, ^14^, ^15^, ^16^, ^17^ Most treatment recommendations are therefore adapted from management of other malignant PanNETs.^2^

In this context, we aimed to describe real‐world management, oncological outcomes, and prognostic factors of patients with VIPoma from a large European multicenter cohort.

## 2 PATIENTS AND METHODS

### 2.1 ENETS database

In 2021, the European Neuroendocrine Tumor Society (ENETS) partnered with the Coordinating Center for Clinical Trials at the Philipps University Marburg (KKS) to establish a new ENETS database (ENETS‐DB) to create a structure to ensure optimal NET patient characterization based on high quality data (www.enets.org). Contracted ENETS centers of Excellence or contracted NET centers, which were certified by the European Reference Networks (ERN), could participate and contribute to the study. In the ENETS‐DB, 250 standardized items (extended level 2) had to be captured, including demographic data (age, sex), hereditary background (presence of MEN1), clinical presentation, time intervals from symptom onset to diagnosis and referral to expert centers, and serum VIP levels, categorized according to multiples of upper limit of normal (ULN). Tumor‐related data comprised primary tumor location, pathological stage according to TNM classification (8th edition), presence and pattern of distant metastases, Ki‐67 proliferation index, and World Health Organisation (WHO) tumor grade.^18^ Imaging modalities, including somatostatin receptor (SRS) imaging, were recorded, where available. Treatment data included surgical interventions (curative and palliative) and clinically relevant postoperative complications classified according to Clavien‐Dindo (CD ≥3,^19^), systemic therapies (SSA, chemotherapy, molecular targeted therapies), peptid receptor radionuclide therapy (PRRT, and loco‐regional liver‐directed treatments (e.g. transarterial (chemo)embolization [TAE/TACE], radiofrequency ablation [RFA]), respectively. For each treatment line, duration, reason for discontinuation, tumor response, and response of hormonal excess were documented, when available. The ENETS‐DB incorporated predefined criteria for hormone‐related clinical response (“functioning syndrome control”). Clinical partial response is defined as a >50% decrease in symptom frequency, while clinical complete response is defined as disappearance of symptoms. Assessment was based on routine clinical evaluation by the treating physicians at the participating centers.

### 2.2 Study design and population

Data from patients with VIPoma, who were treated at participating NET referral centers between July 2004 and December 2024, were included in the ENETS‐DB and retrospectively analyzed. Eligible patients had to fulfill the following criteria: (a) a histologically confirmed PanNET with WDHA syndrome and elevated serum VIP levels exceeding at least twice the upper limit of normal (ULN) and/or immunohistochemically positive tumor staining for VIP; (b) a histologically confirmed PanNET with elevated serum VIP levels exceeding at least twice ULN and any tumor‐related symptoms such as some kind of diarrhea and/or abdominal pain/discomfort. All PanNET diagnoses were performed by expert pathologists in each center, based on neuroendocrine cell morphology and positive immunostaining of at least chromogranin A and synaptophysin. Patients with insufficient clinical or pathological data to confirm VIPoma were excluded. The date of initial VIPoma diagnosis was considered the study baseline. Some clinical data of 23 patients have been published previously.^3^, ^4^, ^20^, ^21^, ^22^

### 2.3 Outcomes

Overall survival (OS) was defined as the time from initial diagnosis to death from any cause. Disease‐free survival (DFS) was calculated from complete resection of VIPoma to first evidence of disease recurrence. The antisecretory efficacy of treatments was assessed by the partial or complete resolution of VIPoma symptoms according to clinical evaluation as documented by the treating physician in the database. Tumor progression could not be analyzed based on central imaging evaluation; thus the duration of antitumor efficacy was measured indirectly as the time to next treatment (TTNT), defined as the time from initiation of one treatment to the date of initiation of the following one (e.g., single agent SSA and then chemotherapy). Combination treatment with an SSA was not considered a treatment change. Patients alive without progression were censored at the date of the last follow‐up (May 08, 2025). Disease status at last follow‐up was categorized as alive with disease (AWD), no evidence of disease (NED), died of disease (DOD), or died of unrelated cause (DURC).

### 2.4 Statistical analysis

Descriptive statistics were used to summarize patient characteristics, tumor features, and treatment patterns. Continuous variables are presented as medians with ranges, and categorical variables as counts and percentages. OS was estimated using Kaplan–Meier curves and expressed as median and 95%‐CI. If estimation of the upper 95%‐CI bound was not possible due to sample size restrictions, the upper bound is presented as NA. Exploratory subgroup analyses were performed according to sex, tumor stage, serum VIP levels, Ki‐67 index, and initial treatment modality. We further divided patients into two time periods based on the date of first treatment, either start of treatment until 2014 (*n* = 31) or after 2014 (*n* = 28). Statistical analyses were descriptive in nature due to the retrospective design and limited sample size. Due to the small sample size and potentially many multi‐collinearities among the available variables, we did not perform multivariable regression. In this exploratory, hypothesis‐generating study, a *p* value of <.05 was considered statistically significant. The statistical software R (version 4.5.0) was used for analysis.

## 3 RESULTS

### 3.1 Patient characteristics

Fifty‐nine of the 70 identified patients had sufficient data and met the diagnostic criteria for VIPoma. Median age at diagnosis was 55  (range 31–84) years, and 32 (54%) patients were male. Five (8%) patients had MEN1. Forty‐six (78%) patients were primarily diagnosed because of a WDHA syndrome, including three MEN1 patients. Another 13 (22%) patients with increased serum VIP levels were diagnosed due to tumor‐associated symptoms such as abdominal pain or discomfort and/or some kind of diarrhea, including two MEN1 patients. Serum VIP concentrations exceeded twice the ULN in 52 patients (88%) and five times the ULN in 30 patients (51%), whereas in 7 (12%) patients the serum VIP at diagnosis was not available. Time from first symptom to diagnosis was a median of 2 (range 0–81) months; time from first symptom to first presentation in a NEN referral center was median 8 (range 0–246) months. In 11 (18%) patients, an initial nonfunctioning PanNET had been transformed into VIPoma after a median of 49 (range 27–163) months (Table 1). Seven of these 11 individuals with secondary development of VIPoma had already distant metastases at the time of PanNET diagnosis, and seven had serum VIP levels >5 × ULN. Time to secondary development of VIPoma did not differ significantly between individuals with VIP levels >5 × ULN compared to individuals with VIP levels <5 × ULN (*p* = .165).

**TABLE 1:** Clinical characteristics of 59 patients with vasoactive intestinal peptide–secreting tumor (VIPoma).

Gender	
Female	27 (46%)
Male	32 (54%)
Age at diagnosis, median (range)	55 (31–84) years
MEN1‐syndrome	5 (8%)
Diagnosis because of	
WDHA syndrome	46 (78%)
Tumor‐related symptoms + VIP >2 × ULN	13 (22%)
Serum VIP >2 × ULN	52 (88%)
Serum VIP >5 × ULN	30 (51%)
Time of first symptom to first positive imaging, median (range)	2 (0–81) months
Time of first symptom to first presentation at a CoE	8 (0–246) months
Transformation NF‐pNEN to VIPoma	11 (18%)
Tumor localized by cross‐sectional imaging	59 (100%)
Localisation VIPoma	
Head	16 (27%)
Body/tail	39 (66%)
Not described	4 (7%)
SRS imaging performed initially	43 (73%)
SRS imaging results (*n* = 43)	
Positive	36 (84%)
Heterogenous	3 (7%)
Negative	4 (9%)
Tumor stage at diagnosis	
Stage I–III	19 (32%)
Stage IV	40 (68%)

Abbreviations: CoE, Center of Excellence; SRS, somatostatin receptor; VIP, vasoactive intestinal polypeptide; WDHA, watery diarrhea–hypokalemia–achlorhydria (WDHA) syndrome.

All 59 (100%) VIPomas were detected in the pancreas by cross‐sectional imaging and 39 of 43 (91%) investigated VIPomas were at least partially positive at initial SRS imaging.

Primary tumors were most frequently located in the pancreatic body or tail (66%) (Table 1).

At diagnosis, 40 patients (68%) had histologically (either biopsy or surgery) confirmed distant metastases (stage IV), and 19 (32%) patients had locoregional (stage I–III) disease. Distant metastases at diagnosis were predominantly confined to the liver (M1a, *n* = 25, 62%) or both hepatic and extrahepatic disease (M1c, *n* = 15, 38%). Fifty‐eight VIPomas were well differentiated and one patient had a NEC. Exact grading with Ki67‐index at diagnosis was available for 51 (86%) of patients. The median Ki‐67 index was 5% (range 1%–40%) and the WHO grade was G1 in 31% (*n* = 18), G2 in 46% (*n* = 27), and G3 in 10% (*n* = 6) of classified patients, respectively. All 51 VIPomas which were analyzed with VIP immunohistochemistry were immunopositive. Thirty‐two (54%) patients underwent primary tumor resection and 16 (50%) of those had tumors >4 cm in size. Lymph node metastases were reported in 11 (34%) of these patients (Table 2).

**TABLE 2:** Pathology of vasoactive intestinal peptide–secreting tumor (VIPOMA) (*n* = 59).

Grading at diagnosis	
G1	18 (31%)
G2	27 (46%)
G3	6 (10%)
NEC	1 (2%)
Well differentiated, but not classified	7 (12%)
Ki67 index, median (range)	5 (1–40)
VIP‐IHC positive	51/51 (100%)
Pathological stage at diagnosis	
I/II	13 (22%)
III	6 (10%)
IV	40 (68%)
Tumor size pathology (32 PT resected)	
T1	1 (3%)
T2	10 (31%)
T3	10 (31%)
T4	6 (19%)
Unclear findings	5 (16%)
Lymph nodes (32 PT resected)	
N0	16 (50%)
N1	11 (34%)
Not defined	5 (16%)
Distant metastases at Dx (*n* = 40)	
M1a: liver only	25 (62%)
M1b: only extra hepatic	0
M1c: both	15 (38%)

Abbreviations: NEC, neuroendocrine carcinoma, PT, primary tumor; VIP‐IHC, Vasoactive intestinal polypeptide immunohistochemistry, Dx, diagnosis

### 3.2 Treatment patterns and outcome

**FIGURE 1:**
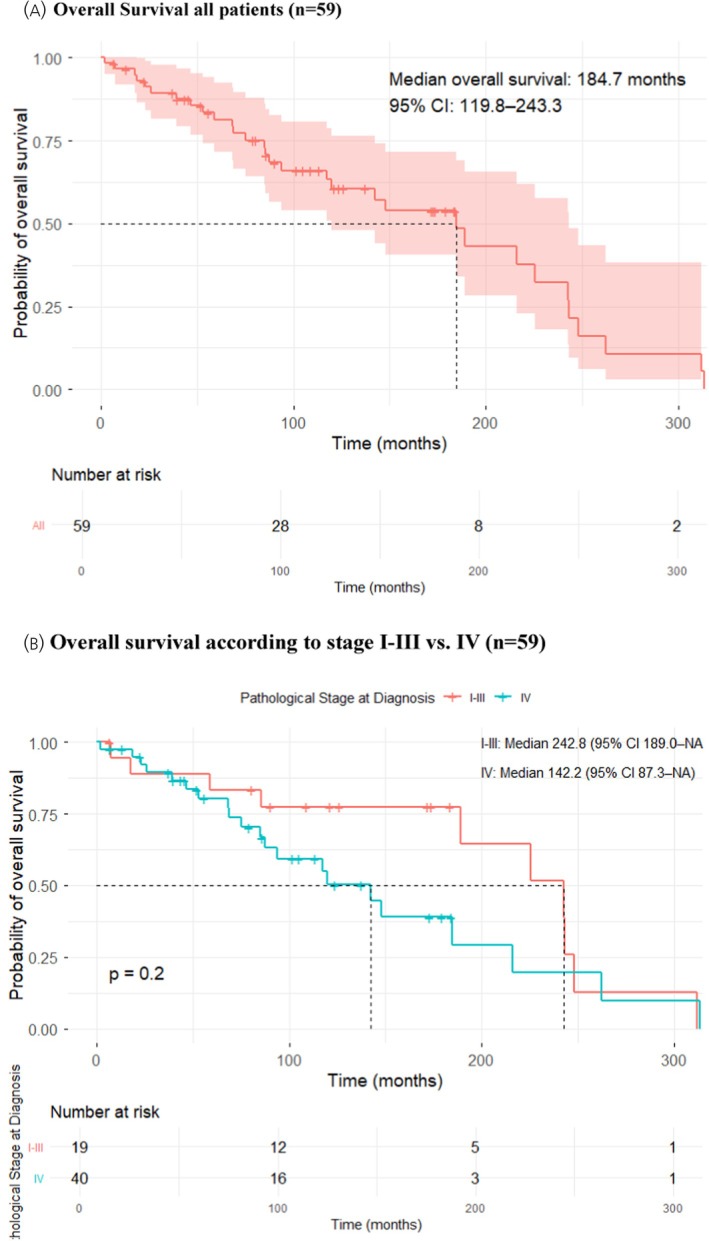
(A) Kaplan–Meier curve for overall survival (OS) in the entire cohort; (B) stratified by stage I–III versus stage IV at diagnosis.

Median OS for the entire cohort was 185 (95%‐CI: 120–243) months; the calculated 5‐ and 10‐years OS were 81% (95%‐CI:72%–93%) and 61% (95%‐CI: 48%–77%) (Figure 1A).

### 3.3 Locoregional VIPoma (stage I–III) at diagnosis (Table 3)

**TABLE 3:** Treatment and outcome in stage I–III vasoactive intestinal peptide–secreting tumor (VIPOMA) (*n* = 19).

0	
Initial curative surgery	14 (79%)
SSA before surgery	1 (5%)
Active surveillance before surgery	1 (5%)
No surgery, SSA only	3 (16%)
Type of surgery (*n* = 16)	
Partial pancreaticoduodenectomy	4
Distal pancreatectomy	11
Total pancreatectomy	1
Resection status	
R0	12 (75%)
R1/R2	0 (0%)
RX	4 (25%)
Complications CD ≥3	
Yes	2 (12%)
No	14 (88%)
Recurrence after R0 resection	10 of 16 (63%)
Median time to recurrence, months	81 (1–215)
Treatment of recurrence	
Re‐resection	3 of 10
SSA	10 of 10
Chemotherapy	7 of 10
Locoregional LDT (TACE, RFA)	3 of 10
Molecular therapy	7 of 10
PRRT	4 of 10
Liver transplantation	2 of 10
Follow‐up, months, median (range)	126 (7–312)
5‐year survival	83% (95%‐CI: 68%–100%)
10‐year survival	78% (95%‐CI: 69%–100%)
Disease status at evaluation date	
AWD	7 (37%)
DOD	7 (37%)
DURC with NED	2 (10%)
Alive with NED	3 (16%)

Abbreviations: AWD, alive with disease, CD, Clavien‐Dindo; DOD, died of disease; DURC, died of unrelated cause; LDT, liver directed therapy; NED, no evidence of disease; PRRT, peptide receptor radionuclide therapy; RFA, radiofrequency ablation; TACE, transarterial chemoembolization.

Surgical resection of the primary VIPoma was performed in 16 of 19 patients with a locoregional confined tumor, most frequently distal pancreatic resections (61%, *n* = 11). Three patients did not undergo surgery; one patient because he refused. All 16 operated patients had R0 or RX (n = 1) resections (Table 3) and all of them had resolution of the hormonal syndrome after resection. Two (12%) patients experienced clinically relevant complications (CD ≥3) after pancreatic surgery. Ten of the 16 (63%) patients who underwent curative intent surgery developed disease recurrence after a median disease‐free interval of 81 (95%‐CI: 1–215) months. Recurrence was treated by various treatment modalities and episodes, most frequently SSA (10/10), molecular targeted therapy (7/10), chemotherapy (6/10), and PRRT (4/10), respectively. In these 10 patients, the first‐line treatment for recurrence was SSA (6/10), molecular targeted therapy (2/10), chemotherapy (1/10), and redo‐surgery (1/10). Of the three patients who did not have surgery, one patient was AWD and two DOD.

After a median follow‐up of 126 (range 7–312) months, 5 patients had NED, but two died of an unrelated cause. Seven patients were AWD and 7 patients DOD. The estimated median OS was 243 (95%‐CI: 189–NA) months with 5‐ and 10‐year survival rates of 83% (95%‐CI: 68%–100%) and 78% (95%‐CI: 69%–100%) (Figure 1B).

### 3.4 VIPoma with distant metastases during the course of disease (*n* = 53) (Table 4)

**TABLE 4:** Treatment and outcome of vasoactive intestinal peptide–secreting tumor (Vipoma) stage IV at diagnosis or distant metastases at any time (*n* = 53).

Treatment modality	Pat. receiving treatment (*n* = 53)	Treatment episodes (*n* = 354)
Surgery	31 (58%)	52 (15%)
Surgery of the primary stage IV at Dx	16 (52%)	16 (31%)
Surgery of liver metastases	11 (35%)	13 (25%)
Surgery of peritoneal metastases	2 (6%)	2 (4%)
Liver transplantation	4 (13%)	4 (8%)
Other surgery	6 (19%)	7 (13%)
SSA	53 (100%)	77 (22%)
Chemotherapy	32 (60%)	69 (19%)
Alkylating‐based	24 (72%)	32 (46%)
Platin based	20 (55%)	23 (33%)
Other regimens (e.g., FOLFIRI)	11 (31%)	12 (17%)
Molecular targeted therapy	29 (55%)	53 (15%)
Sunitinib	24 (83%)	27 (51%)
Everolimus	15 (52%)	16 (30%)
Other (e.g., Cabozantinib, Sorafenib)	7 (21%)	10 (19%)
PRRT	23 (43%)	31 (9%)
Locoregional LDT	24 (45%)	72 (20%)
First line treatment		
SSA	30 (57%)	
Surgery	17 (32%)	
Chemotherapy	3 (6%)	
Other (e.g., Bevacizumab)	3 (6%)	
Follow‐up stage IV at Dx, months, median (range)	82 (2–313)	
5‐year survival	80% (95%‐CI: 68%–95%)	
10‐year survival	50% (95%‐CI: 35%–73%)	
Disease status stage IV at Dx (*n* = 40)		
AWD	19 (48%)	
DOD	16 (40%)	
DURC with disease	5 (13%)	

Abbreviations: AWD, alive with disease; DOD, dead of disease; DURC, dead of unrelated cause; Dx, diagnosis; LDT, liver directed therapy; PRRT, peptide receptor radionuclide therapy; RFA, radiofrequency ablation; TACE, transarterial chemoembolization.

Of 53 patients, 40 had distant metastases at initial diagnosis and another 13 developed distant metastases during the course of disease. Patients with distant metastatic disease received a median number of six (range 1–19) therapeutic lines during the disease course. Six (15%) patients with stage IV VIPoma at diagnosis underwent primary tumor resection with curative intent (R0) concomitant resection of liver metastases, resulting in a median DFS of 90 months (95%‐CI: 23–NA) and an OS of 142 months (95%‐CI: 142–NA). In addition, four (8.3%) patients had a potential curative liver transplantation 83 to 130 months after initial diagnosis and 4–6 different treatment modalities before transplantation, resulting in a DFS of 16 to 192 months. The median OS of transplanted patients varied from 126 to 313 months; two patients were AWD at data‐base cut‐off date (Table S1).

All patients with distant metastatic disease received palliative therapy. Overall, 354 treatment lines were given to 53 patients (Table 4). SSAs were received most frequently (100%), followed by systemic chemotherapy (60%), surgical interventions (58%), molecular targeted therapy (55%), liver‐directed therapy (45%), and PRRT (43%), respectively. The main first‐line treatments used were SSA (57%) and surgery (32%). In patients with complete data, SSA, chemotherapy, molecular targeted therapy, PRRT, and locoregional liver‐directed therapy led to a complete or partial response of the VIPoma syndrome in 62% (35/56), 64% (16/25), 62% (15/24), 71% (15/21), and 66% (35/53), respectively (Table 5). Systemic treatment had to be stopped because of toxicity in 1% (1/74) of patients receiving SSA, in 15% (10/67) receiving chemotherapy, and in 22% (11/51) receiving molecular therapy, and 3% (1/31) receiving PRRT, respectively. All lines combined, the longest median TTNT were observed after liver transplantation (31 months), PRRT (21 months), and any resective surgery (17 months), whereas molecular targeted therapy had the shortest TTNT (6 months) (Table 5).

**TABLE 5:** Type and time to next treatment (TTNT) in patients with distant metastatic vasoactive intestinal peptide–secreting tumor (VIPoma) (whatever therapeutic line) (*n* = 53).

Treatment	Patients (*N*)	Treatment episodes	CR/PR of VIPoma syndrome^a^	TTNT (months, median (range))
SSA	53	77	62% (35/56)	9 (0–147)
Chemotherapy	32	69	64% (16/25)	11 (1–132)
Molecular targeted therapy	29	53	62% (15/24)	(1–68)
Surgery, any resection	31	48	Not evaluable	17 (0–151)
Locoregional LDT	24	72	66% (35/53)	9 (0–49)
PRRT	23	31	71% (15/21)	21 (1–102)
Liver transplantation	4	4	No data	31 (16–191)

Abbreviations: a, with regard to treatment episode, if documented; CR/PR, complete/partial response; LDT, liver‐directed therapy; PRRT, peptide receptor radionuclide therapy; SSA, somatostatin analogues.

The estimated OS of patients with stage IV VIPoma at diagnosis was 142 (95%‐CI: 87–NA) months with 5‐ and 10‐year survival rates of 80% (95%‐CI: 68%–95%) and 50% (95%‐CI: 35%–73%). The disease‐specific 5‐ and 10‐year survival rates were 80% (95%‐CI: 68%–95%) and 67% (52%–85%). After a median follow‐up of 82 (range 2 to 313) months, 19 (48%) patients were AWD, 16 (41%) had DOD, and 5 (13%) had DURC.

### 3.5 Prognostic factors

**FIGURE 2:**
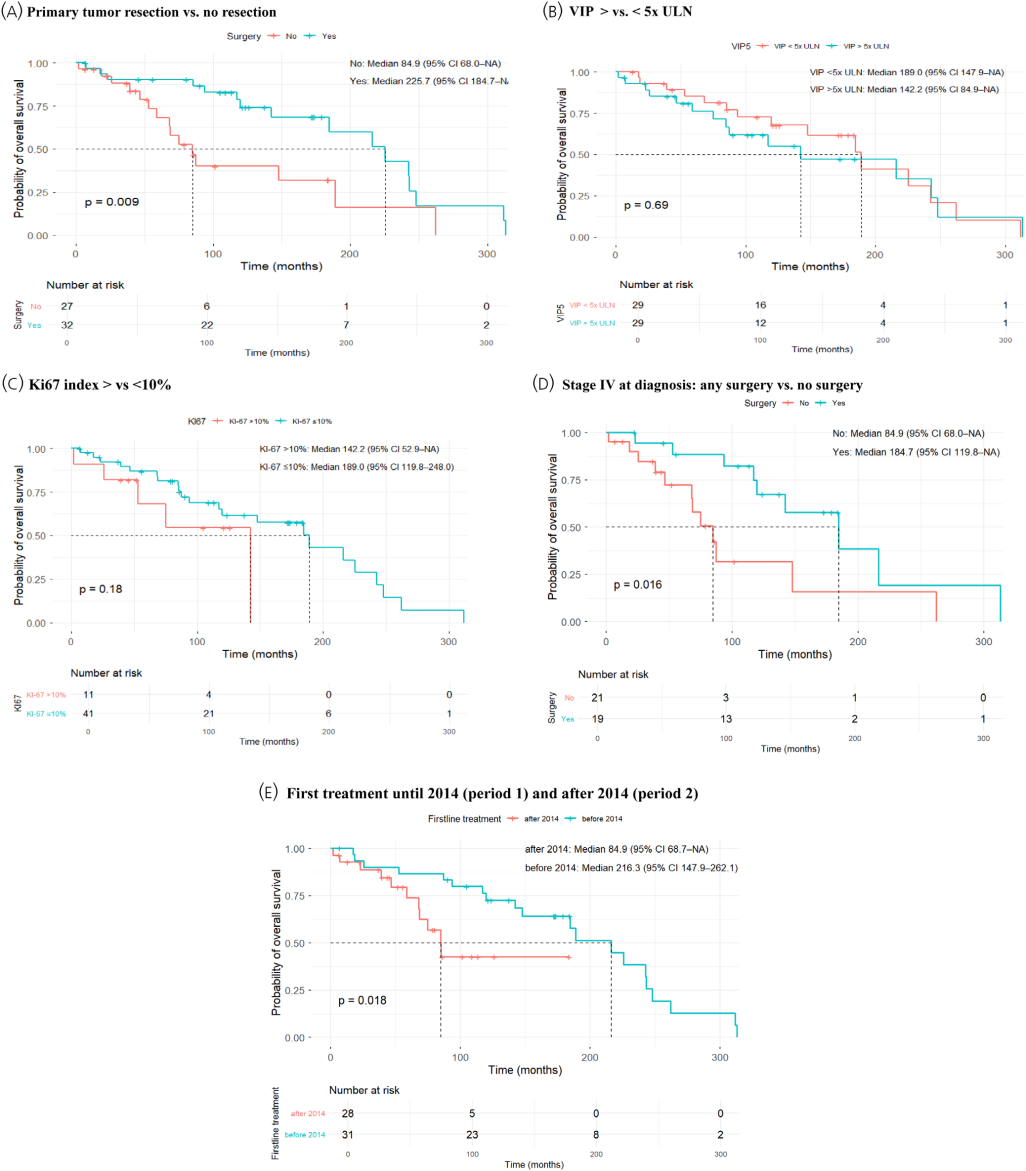
Prognostic factors. Overall survival (OS) stratified by (A): Surgery versus no surgery, (B): Serum VIP </>5 × ULN; (C): Ki67 index </>10%; (D): Stage IV at diagnosis: Any surgery versus no surgery; (E): Initial treatment until 2014 versus after 2014.

Univariate assessment of prognostic factors (e.g., sex, stage at diagnosis, serum VIP levels, Ki67 index) in the whole cohort (*n* = 59) only showed a statistically significant difference for primary tumor resection. Patients who underwent primary tumor resection versus those without had a significantly longer median OS (216 vs. 85 months, *p* = .009, Figure 2A). Other parameters did not reach statistical significance. This was noted for the OS of female versus male patients (225 vs. 143 months, *p* = .057), patients with stage I–III versus stage IV (243 vs. 142 months, *p* = .2, Figure 1B), serum VIP <5 versus >5 × ULN (189 vs. 142 months, *p* = .69, Figure 2B) and Ki67‐index <10% versus >10% (189 vs. 142 months, *p* = .18, Figure 2C), respectively. Surprisingly, first treatment until 2014 (period 1, *n* = 28) versus after 2014 (period 2, *n* = 31) showed a decreased median overall (85 vs. 216 months, *p* = .018) and progression‐free survival (68 vs. 120 months, *p* = .019) in period 2 (Figure 2E). However, there was a higher censoring rate in period 2 (61% vs. 35%) and a shorter median follow‐up time (median 57 months vs. 148 vs., *p* < .001). In addition, patients in period 2 were significantly older at diagnosis (median 51 years vs. 58 years, *p* = .03). It is also of note, that in period 1 71% (22/31) of patients underwent surgery compared to only 36% (10/28) in period 2 (*p* = .003) in period 2, whereas 79% (22/28) of patients were initally treated with SSA in period 2 versus 32% (10/31) in period 1 (*p* = .001). In patients with stage IV disease at diagnosis, the only significant prognostic factor was surgery. Patients with stage IV disease who underwent any surgical resection (*n* = 19, of those 16 with resection of the primary tumor) had a significantly longer median OS (185 vs. 85 months, *p* = .016) compared to patients who had no surgery (*n* = 21) (Figure 2D).

## 4 DISCUSSION

To the authors' knowledge, this is the largest series of VIPomas, which were previously reported in detail in case reports or retrospective cohort studies with up to 31 patients.^3^, ^4^, ^6^, ^11^, ^12^, ^14^ Data on 59 patients with a VIPoma diagnosis were collected from certified NET referral centers (treated over a 20‐year period), accounting for an estimated less than 0.5% of newly diagnosed GEP‐NET in these centers, given the number of requested newly diagnosed 100 GEP‐NET cases/year to be certified as an ENETS center of excellence. Thus, VIPoma is probably nowadays even rarer than the 1%–3% prevalence reported.^2^, ^23^ Due to its rarity, no clear diagnostic criteria exist to define VIPoma. In the present series, we applied strict criteria to document the diagnosis. Diarrhea alone might be a non‐specific criterion to define VIPoma, since other PanNET‐related functioning syndromes can cause diarrhea as well as SSA treatment.^24^ A recent international study also reported that elevated VIP concentrations alone are not predictive for a VIPoma and most patients with elevated VIP do not have a VIPoma.^25^ Among 70 patients identified, 11 (15%) were excluded despite clinical suspicion of VIPoma, because they did not reach the full diagnostic criteria. When applying the criteria used in the present study, about 10% of included patients in previously published retrospective VIPoma series would have been excluded.^6^, ^11^, ^14^ In the present series, we took advantage of standardized documentation in the new ENETS‐DB (www.enets.org) with approximately 250 items to record clinical characteristics and management, including novel treatments such as molecular targeted therapy and PRRT, as well as treatment outcome.

### 4.1 Clinical presentation and tumor characteristics

Consistent with previous reports, VIPoma predominantly affected middle‐aged adults and showed a slight male predominance.^3^, ^4^, ^11^, ^12^, ^13^ More than half of tumors were larger than 4 cm in size and two‐thirds of patients presented with distant metastases at diagnosis, underscoring the aggressive clinical behavior and frequent diagnostic delay associated with this disease.^3^, ^4^, ^6^ The high rate of distant metastases at diagnosis is in line with most previous reports ranging from 60%–78%.^3^, ^4^, ^11^ The predominance of tumors arising in the pancreatic body and tail observed in the present cohort mirrors previous series.^3^, ^4^, ^6^, ^12^ In the present series, 8% of tumors were associated with the MEN1 syndrome, which is lower than in recent French studies with 11% to 13.6%.^4^, ^5^ Of note, in the present series, 18% of VIPomas were initially diagnosed as non‐functioning and underwent functional transformation over time, as reported in one other series.^12^

Fifty‐eight (98%) patients had well differentiated tumors; of those at least 58% were classified as WHO grade G2 or G3 tumors, leading to substantial aggressiveness. The high proportion of G2/G3 tumors was already reported in VIPoma, as well as in other functioning PanNET.^4^, ^26^

### 4.2 Locoregional disease

Management of these patients presents the dual challenge of controlling tumor progression and/or the hormonal syndrome. In patients with locoregional (stages I–III) VIPoma, complete (R0) surgical resection may meet both objectives and is recommended by current guidelines.^2^, ^8^ In the present series, 32% of patients had stage I–III tumors at diagnosis, which is in the range of 23% and 40% in previous series.^3^, ^4^, ^6^ Surprisingly, only 16 patients underwent potential curative resection, whereas three patients underwent SSA treatment. In the present series, all patients with resected stage I–III VIPoma were free of symptoms after surgery, mirroring the experience of previous reports.^3^, ^4^, ^10^, ^11^, ^12^, ^13^, ^14^, ^15^, ^16^, ^17^ However, 63% experienced recurrence, which is in line with previously reported recurrence rates of 66% to 80%.^3^, ^4^ This is a relatively high recurrence rate compared to other functioning PanNET, such as gastrinoma (~30%–60%), glucagonoma (~30%–50%) and malignant insulinoma (~20%).^26^, ^27^, ^28^ The median recurrence‐free survival in the current cohort was 87 months, which is higher than the reported 20 and 45 months in other series.^3^, ^4^ The 5‐year survival rate in the present series was 83% compared to 94% in an older literature review.^29^ Despite the high frequency of recurrence after resection, long‐term survival could be achieved, supporting an aggressive surgical approach in patients with locoregional disease at diagnosis.^2^, ^8^

### 4.3 Distant metastatic disease

Although more therapies for metastatic PanNET have become available during the last decade, very limited data have been reported regarding their efficacy and oncological outcome in patients with VIPoma. For patients with distant metastases at diagnosis, complete tumor resection was possible in only 15% of patients, which is lower than the 40% in the previous French series.^4^ Curative‐intent surgery also yielded effective and relatively durable disease control with median DFS of 90 months. Therefore, consideration of potentially curative intent surgical resection of a VIPoma and associated metastases should always be discussed in a multidisciplinary tumor board.^2^, ^8^, ^9^

Patients with unresectable distant metastases had undergone a median of 6 (range 1–19) treatment modalities during the course of their disease, with a total of 354 treatment episodes. The only other study providing detailed treatment data reported 87 treatment episodes in 21 patients,^4^ including most treatments nowadays available.^2^, ^20^, ^30^, ^31^, ^32^, ^33^, ^34^ Of note, compared to all other previous series^2^, ^3^, ^4^, ^6^, ^10^, ^11^, ^12^, ^13^, ^14^, ^15^, ^16^, ^17^, ^20^, ^29^, ^30^, ^31^, ^32^, ^33^, ^34^ SSAs were received most frequently (100%). According to the ENETS guidelines,^2^ SSA is usually the recommended first‐line therapy in patients with metastatic VIPoma for its antitumor and antisecretory efficacies, especially in patients with G1 or low G2 tumors. Accordingly, in the present series, SSA was given first‐line in 60% of patients (Table 4). However, similar to the French cohort,^4^ the durability of SSA monotherapy appeared limited in the present cohort, with additional systemic or locoregional treatments frequently needed. This highlights the need for close follow‐up and early treatment optimization in case of VIPoma progression.

According to ENETS and ESMO guidelines,^2^, ^9^ systemic treatments with chemotherapy or PRRT should be considered in patients with high G2 or G3 tumors or those with significant tumor burden, because they allow both control of hormonal symptoms and obtain a greater antitumor response. Thus, it is surprising that only 6% of patients received chemotherapy first‐line and none PRRT, although almost 60% of patients had G2/G3 tumors. This is much lower than 35% and 33% reported chemotherapy rates in the French GTE and Greek series.^3^, ^4^ Alkylating‐based regimens are the chemotherapy with the highest level of evidence in PanNETs^2^ and were thus used in the present cohort in 72% of patients.

PRRT was used in 48% of patients, accounting for 9% of treatment episodes. In contrast, in the French GTE cohort, PRRT was used in only 4%, accounting for 1% of treatment episodes. As in other studies,^21^, ^34^ we noted good antitumor effect in VIPoma, with very low short‐term toxicity (3%) compared to chemotherapy (15%), and molecular targeted therapy (22%). This may support a more frequent use of PRRT in patients with a VIPoma diagnosis, especially since 79% to 84% of these tumors are positive on SRS‐imaging.^3^

Data regarding molecular targeted therapy, including everolimus and sunitinib, in VIPomas are scarce. Data in the literature have reported a fast and good effect of sunitinib in patients with metastatic VIPomas.^20^, ^30^ This could be confirmed in the present series, although the positive effect lasted only for a limited time. In the French VIPoma series^4^ sunitinib seemed to provide a better combination of antisecretory and antitumor efficacy than everolimus, which could not be confirmed in the present series.

Locoregional liver‐directed therapies (LDTs), such as transarterial [chemo]‐embolization, percutaneous radiofrequency ablation, or microwave ablation, can be efficient treatment options for patients with liver predominant disease.^2^, ^3^, ^4^, ^31^, ^32^, ^33^ This is confirmed in the present cohort study, as 66% of patients undergoing locoregional LDT showed complete or partial response of the hormonal syndrome for a certain time.

Although liver transplantation was performed in only four (8%) patients with metastatic VIPoma in the present cohort, this is a high proportion compared to other reported studies on **f**unctioning PanNETs. To our knowledge, only nine prior case reports on liver transplantation for VIPoma, including one of our patients,^22^ have been published. In 2011, a systematic review reported data on 61 patients with functioning pancreatic NET who underwent liver transplantation; among those, five had VIPoma.^35^ The authors concluded that the prognosis following liver transplantation for VIPoma appeared to be more favorable compared to other NET diagnoses. Notably, all five patients were alive at post‐transplantation intervals ranging from 12 to 123 months, with a recurrence‐free survival at 5 years of 47%.^22^ The longest survival after liver transplantation reported for metastatic VIPoma so far was 9 years.^36^ We can confirm these favorable results with an OS after transplantation of 29–206 months. Thus, liver transplantation should be more often considered in selected patients with metastatic VIPoma.^36^, ^37^

While measurement of PFS would have been preferable, morphological progression due to imaging was not measurable in this cohort, thus antitumor efficacy was indirectly assessed by measurement of TTNT as in a previous study on glucagonoma.^38^ All lines combined, the longest median TTNT was observed with liver transplantation (31 months), PRRT (21 months), and surgery (17 months), respectively. In a French study,^4^ chemotherapy, everolimus, and sunitinib achieved a median PFS of 9, 11, and 12 months respectively, whereas it was not reported for surgery and none of the patients was transplanted.

### 4.4 Prognostic factors

A recent French study^4^ reported a poorer prognosis when distant metastases were present at diagnosis, but the OS curve difference did not reach statistical significance (*p* = .055), as in the present series (Figure 1B). Moreover, higher baseline plasma VIP concentrations (*p* = .025) and higher Ki67 indexes (*p* = .045) were significantly associated with shorter OS in this study.^4^ While the present study showed the same direction in difference, statistical significance was not reached, most likely due to high censoring rates and partially very long survival rates in both groups respectively, leading to crossing Kaplan–Meier curves.

The only significant positive prognostic parameters in the present study were treatment in period 1 (before 2014) versus period 2 (after 2014, ***p* = .018**) and primary tumor resection versus no primary tumor resection (*p* = .009) and any surgery versus no surgery in stage IV patients (*p* = .016). The surprising better overall and progression‐free survival in period 1 might be explained by the higher censoring rate in period 2 (61% vs. 35%) as well as significantly shorter follow‐up (57 vs. 148 months, *p* = .001). It might, however, somehow indicate that a more aggressive attitude with initial surgery of VIPoma might lead to a more favorable outcome compared to initial SSA treatment.

### 4.5 Limitations

Although this is the largest clinical series reported so far, one limitation remains: the limited number of patients. The retrospective design of this study is another inherent limitation, including some missing data and heterogeneity in diagnostic and therapeutic approaches across centers over the 20‐year period. The relatively low patient number precluded robust multivariable analyses and limits the generalizability of subgroup comparisons. Nevertheless, given the scarcity of prospective data, large retrospective cohorts— such as the presented study— provide essential insights into real‐world management and outcomes.

## 5 CONCLUSIONS

VIPoma is an extremely rare but clinically challenging functioning PanNET, characterized by severe symptoms and a high rate of metastatic disease at diagnosis. Treatment decisions should be made by a multidisciplinary tumor board at expert centers. Multidisciplinary management in expert centers provides long‐term survival, although cure is rare. Since the management of distant metastatic disease is quite heterogeneous, collaborative prospective studies are needed to define optimal treatment algorithms.

## AUTHOR CONTRIBUTIONS

**J. Hofland:** Data curation; writing – review and editing. **L. de Mestier:** Data curation; writing – review and editing. **L. Mais:** Data curation; writing – review and editing. **E. Tiensuu Janson:** Writing – review and editing; data curation. **E. Baudin:** Methodology; writing – review and editing; data curation. **G. Kaltsas:** Data curation; writing – review and editing. **Bartsch D K:** Conceptualization; writing – original draft; writing – review and editing; investigation; data curation; methodology; validation; formal analysis. **F. Spada:** Writing – review and editing; data curation. **M. G. McNamara:** Writing – review and editing; data curation. **H. Leupe:** Writing – review and editing; data curation. **R. Garcia‐Carbonero:** Writing – review and editing; data curation. **M. Andreassen:** Writing – review and editing; data curation. **S. Grozinsky‐Glasberg:** Writing – review and editing; data curation. **F. Panzuto:** Data curation; writing – review and editing. **H. Klinkhammer:** Formal analysis; writing – review and editing; methodology; validation. **M. Fleschen:** Methodology; software; project administration.

## CONFLICT OF INTEREST STATEMENT

Johannes Hofland reports speaker or consultation fees from Ipsen, Novartis, and Serb. The remaining authors declare no conflicts of interest related to this work.

## ETHICS STATEMENT

This study was performed according to the World Medical Association Declaration of Helsinki. Data collection was anonymized prior to analysis, and the ENETS‐DB was approved by the Ethic Committee of the Philipps‐University Marburg (No. 187/21), as well as all participating centers. All living patients were informed and gave their consent for the collection of their pseudonymized data.

## Supporting information


**Table S1.** Clinical characteristics of VIPoma patients with liver transplantation (*n* = 4).

## Data Availability

The data that support the findings of this study are available on request from the corresponding author. The data are not publicly available due to privacy or ethical restrictions.
