# Supplementary material for: Characteristics, management, and outcomes of patients with VIPoma–A retrospective analysis of the ENETS database
Source: J Neuroendocrinol. 2026 Jun 23;38(7):e70219. doi: 10.1111/jne.70219 (PMC13291538; doi:10.1111/jne.70219)
Supplement: Supplementary file 1 — Table S1. Clinical characteristics of VIPoma patients with liver transplantation (n = 4). [file JNE-38-e70219-s001.docx]

**Table S1: Clinical characteristics of VIPoma patients with liver transplantation (n=4)**

| **Patient** | **Age at Dx, years, gender** | **Stage at Dx, grading** | **Initial Treatment** | **Treatments before LTX** | **Time between initial Dx and LTX, months** | **Follow-up after LTX, months** | **Recurrence after LTX** | **Time to recurrence, mo.** | **Total follow-up, mo.** | **Disease status** |
| --- | --- | --- | --- | --- | --- | --- | --- | --- | --- | --- |
| **1** | 38, male | IV, G2 | TAE | TAE  SSA  PRRT,  TACE  PRRT | 83 | 59 | yes | 28 | 142 | DOD |
| **2** | 33, female | II, G2 | Surgery curative | Curative Surgery Surgery recurrence,  RFA Liver mets.  Chemotherapy  PRRT | 98 | 29 | yes | 16 | 126 | AWD |
| **3** | 55, female | IV, G2 | SSA | Debulking  Chemotherapy  Interferon  RFA liver mets. | 107 | 206 | yes | 192 | 313 | DOD |
| **4** | 39, male | II, G1 | Surgery palliative | RE-Debulking  SSA  MTT  Chemotherapy  MTT | 130 | 47 | yes | 34 | 172 | AWD |

TAE- transarterial emblization; SSS- somatstatin analogues; PRRT- peptide receptor radionuclide therapy; RFA- radio frequency ablation; MTT- molecular targeted therapy
